# Non Destructive Defect Detection by Spectral Density Analysis

**DOI:** 10.3390/s110302334

**Published:** 2011-02-24

**Authors:** Ondrej Krejcar, Robert Frischer

**Affiliations:** 1 Department of Measurement and Control, CAK, FEECS, VSB Technical University of Ostrava, Ostrava, Czech Republic; 2 Department of Automation and Computing in Metallurgy, VSB Technical University of Ostrava, Ostrava, Czech Republic; E-Mail: robert.frischer@vsb.cz

**Keywords:** FFT, power spectrum, MatLab, Statistica, defect

## Abstract

The potential nondestructive diagnostics of solid objects is discussed in this article. The whole process is accomplished by consecutive steps involving software analysis of the vibration power spectrum (eventually acoustic emissions) created during the normal operation of the diagnosed device or under unexpected situations. Another option is to create an artificial pulse, which can help us to determine the actual state of the diagnosed device. The main idea of this method is based on the analysis of the current power spectrum density of the received signal and its postprocessing in the Matlab environment with a following sample comparison in the Statistica software environment. The last step, which is comparison of samples, is the most important, because it is possible to determine the status of the examined object at a given time. Nowadays samples are compared only visually, but this method can’t produce good results. Further the presented filter can choose relevant data from a huge group of data, which originate from applying FFT (Fast Fourier Transform). On the other hand, using this approach they can be subjected to analysis with the assistance of a neural network. If correct and high-quality starting data are provided to the initial network, we are able to analyze other samples and state in which condition a certain object is. The success rate of this approximation, based on our testing of the solution, is now 85.7%. With further improvement of the filter, it could be even greater. Finally it is possible to detect defective conditions or upcoming limiting states of examined objects/materials by using only one device which contains HW and SW parts. This kind of detection can provide significant financial savings in certain cases (such as continuous casting of iron where it could save hundreds of thousands of USD).

## 1. Introduction

**Figure 1.**
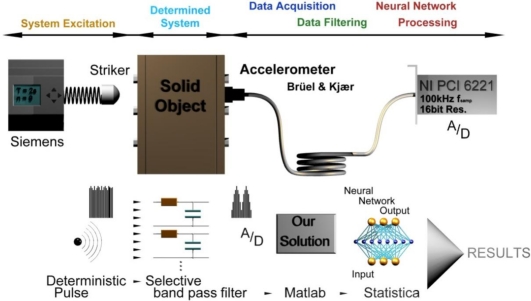
Schema of measuring network and procedure of data process. All operations are performed on a physical model of a crystallizer.

The field of technical diagnosis is very extensive. A device’s maintenance and economical performance is dependent on it. This is the reason why it is important to know the actual condition of an object (device) or the condition of its significant components. It is often impossible to determine the actual parameters of certain devices because shut-down would be uneconomical (for example, devices for continuous casting of steel, high-pressure gas pipes, or just simple engines which would have to be taken apart to detect cylinder failure). Technical diagnosis provides an intuitive, empirical and systematic approach to the maintenance of certain objects without taking them apart or suspending their activity. Technical diagnosis correctly done increases the reliability and safety of a device. The basic function of the diagnosis is to determine the critical spots of a monitored object. We could try to install a physical quantity sensor in these spots, which would characterize a defect or its emergence. At this point we face many challenges. The placement of sensors is often impossible because of the movement of parts (either a linear shift or rotational motion). Additionally, surface crack sensors whose function on the basis of magnetic fields are very complex and expensive and they require a specific adjustment for each type of setting. Especially particular clefts are critical for the majority of objects and prompt detection is crucial for the safe operation and economical repair. This is the reason why the nondestructive diagnosis option is an important emerging discipline. A scheme of the measurement chain is show in Figure 1.

## 2. Related Work

This article is devoted to the technical diagnostics of a device using analysis and evaluation of its vibration spectrum or acoustic emissions. The vibration spectrum and acoustic emissions have various origins. When analyzing a vibration spectrum, the response of a system (of an object) to an artificially created impulse is recorded. On the other hand, acoustic emissions mainly originate spontaneously, for example, by creating a crack on a pipe’s surface during mechanical stressing (in plastic deformation position), *etc.*

On the basis of the problem definition provided by an external company, the task was to find out whether it is possible to detect the internal conditions of an object using vibration spectrum analysis. The object consisted of a metallic skeleton connected by screws placed on the edges (Figure 1). When a screw is loosened, the diagnostic system should define precisely the position or side where the screw (or simply the defect) is located. Many articles have been published considering this topic, [1–4], but most of them address specific problems and this translated into very specialized solutions.

**Figure 2.**
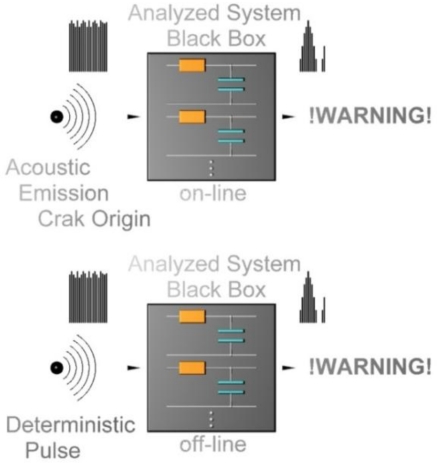
The upper part shows online identification. The bottom figure shows our method of offline condition identification.

For example, in [4] the authors were interested in the problem of crack formation in rotor and turbine blades. Their method involves the detection and analysis of acoustic emissions. The main difference between their and our solution is that while theirs was trying to discover an already evolved defect (crack), our solution enables detection of emerging cracks, because it uses another source of vibrations (deterministic pulse). Whilst the formation of acoustic emissions is controlled in [4] (passive method), our method is aimed at recording an enforced response. The differences can be illustrated by the image shown in Figure 2.

The main advantage of our solution is the possibility of preventing the formation of limiting states and also the prevention of dangerous situations and device damage. Whilst the authors of the aforementioned article are trying to preclude greater damages after the formation of a crack, our option tries to prevent the condition from happening. A comparison of both approaches may be seen in Table 1.

A third option of analysis with the assistance of acoustic emissions is mentioned in the Table, however it differs from that presented in [4] by being mobile. It relies in the application of software which is designed for mobile devices This solution would enable its use, for *i.e.*, detection of engine defects in automobiles or electrical engines while applying acoustic emission analysis through the microphone input of a mobile device. The range of recorded frequencies of the input is typically up to 22 kHz, which is sufficient for that usage. However, this article does not discuss this solution, which will be approached in the future.

## 3. Experimental Section

### 3.1. Analysis of the Response of Deterministic Pulse

**Figure 3.**
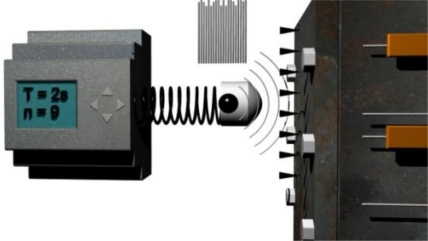
Pseudo Dirac impulse realized by a firing pin which is controlled by a Siemens PLC. T is the pulse period and n is a number of pulses.

The principle of this method comes from measuring a system’s (examined object’s) response to a Dirac impulse. The first experimental results were presented in [5]. An ideal Dirac impulse is replaced by a real impulse which is generated by a firing pin that is excited by a magnetic field (Figure 3).

**Figure 4.**
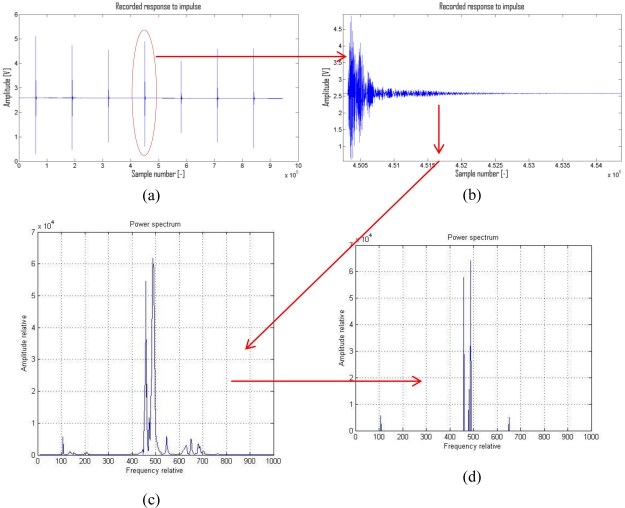
(**a**) Record series of measurements. (**b**) Amplitude envelope of chosen response on pulse. (**c**) High-performance spectral density of given pulse without any other modification. (**d**) Modified high-performance spectral density. The suggested filter was used in a Matlab environment.

This impulse is far from the ideal shape, but for these purposes it is sufficient. Individual pulses are operated by a Siemens PLC which generates a series of impulses with a period of 2 s (Figure 3). Unwanted offset and relatively uneven progress of individual strokes are among the basic disadvantages (only when comparing amplitude envelope). A number of measurements is made progressively aimed at eliminating these defects. At least four out of nine of these measurements are very similar and the others show moderate or extreme anomalies or periodic defects (vibrations from surroundings, noise from amplifier and so forth). A digital record is made for the whole series of measurements at once. The records of individual pulses are then extracted from the whole and saved independently [Figure 4(a,b)].

An impulse is a source of vibrations (or also specific acoustic emissions [6]) with a wide range of frequencies (spectral density [6]). All of the components of the whole spectrum would be represented in an ideal pulse (theoretically a white noise generator). The components up to several kHz are represented in our case. Moreover, there is uneven representation of individual components. An analyzed object behaves as a selective band-pass filter [6] after the activation of pulse or after the formation of an acoustic emission. The recorded amplitude‘s envelope is modified and the FFT (Fast Fourier Transform) [Figure 4(c)] is applied.

**Figure 5.**
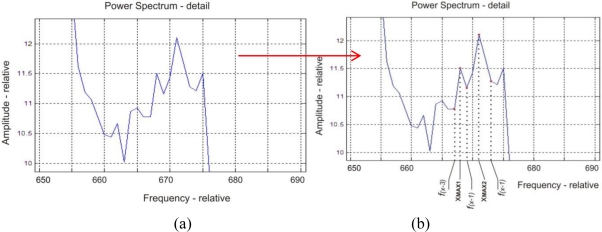
**(a)** Detail of point in PS. **(b)** False maximum during application of simple algorithm for finding maximum.

The final power spectrum density (PSD) is then subjected to examination and modification. Some vibrations (at certain frequencies) pass without significant changes, some are heavily suppressed. In order to work more synoptically with the received data, it is necessary to modify the PSD. After the application of the suggested filter, the irrelevant data are removed from the PSD and the result is saved in a matrix form [Figure 4(d)]. The suggested filter scans each pulse record (its PSD) and searches for specific values of the individual spectrum’s components. Positions of points (their corresponding frequencies) are very important for the next analysis of the PSD. It is quite difficult to find these anomalies, because the signal spectrum has an odd shape [Figure 5(a)]. Hundreds of data which did not correspond to the distribution of maximums in the spectrum were received after the application of classic algorithm for finding the maximum (f_(x−1)_ < max > f_(x+1)_). While searching for the cause of algorithm’s erroneous functioning, a simple cause was discovered. It is clear after enlarging part of the curve that it is not smooth. Modulated points created a number of false (pseudo) maxima [Figure 5(b)] which must be eliminated.

The newly proposed filter (described further in the article) was able to eliminate these pseudo maxima. It went through the record and for the highest value in a certain area (local maximum) it verified whether it is really the highest. This interval is optional and its value is inversely related to the number of maxima in the record. Moreover, this interval may be expressed as insensitivity. Its value tells us in which interval a certain local maximum must be valid in order for its position to be clarified and saved. Furthermore, in order to remove the ubiquitous noise from the signal, the local maxima with amplitude smaller than 7% of the global maximum were eliminated. In this way the image was cleaned and sent to the next processing step with the assistance of a neural network. Work on the filter used is still in progress and by its improvement it is possible to achieve even better results.

## 4. Results and Discussion

**Figure 6.**
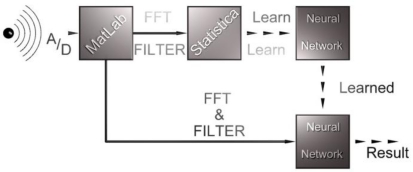
Schematic of the measurement network.

A schematic of the measurement series is depicted in Figure 6. Vibrations are scanned with the assistance of a type 4332 accelerometer from Bruel & Kjaer. This sensor is unique for its high frequency range, which exceeds 25 kHz. Indeed, there are cheaper types of accelerometers from various companies on today’s market, but most of them are only suitable for scanning frequencies up to 600 Hz (mobile applications) which is not convenient for the declared purposes. On the other hand, they might be used in applications that do not require high scanning frequencies, because they are often implemented in modern smartphones or PDA devices.

After the signal amplification (the sensor only produces tens of mV) the signal is digitalized with the help of a NI PCI 6221 multi I/O card from National Instrument. This card has 16 analog inputs which run at 250 kHz. Only one channel that works with a sample frequency of 100 kHz was used for the given purpose. With regard to the estimated and scanned frequencies on the order of tens of kHz (maximum around 20 kHz) fivefold oversampling is sufficient. A driver algorithm was created in the Matlab—Simulink environment and adjusted in such a way that the trapped data are saved for a period of 20 s. The subsequent processing of results was already running offline.

The algorithm is evaluated in Matlab. Firstly, it is necessary to extract relevant data, records of pulses and save them separately into a matrix. A routine serves for this purpose. It looks for initiation of pulses and then saves identically long blocks of data into the beforehand set positions in the matrix:

(1) $${\mathrm{start}}_{i}=\mathrm{if}(\mathrm{abs}(\mathrm{DATA}(j)-\mathrm{DATA}(j+1))>(\mathrm{noise.level}\cdot \sigma )$$

where:

DATA: is a matrix with the record of pulses
i: index of variable start, sequential number
j: sequential number of sample in the record
σ: is a constant derived from medium level of noise in signal and states that: σ > 2

**Figure 7.**
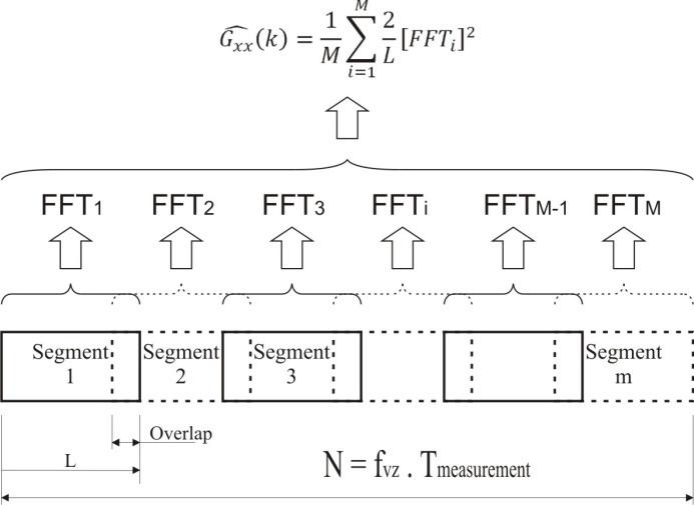
Welch’s method of modified periodograms.

After extraction of individual records, a Fast Fourier Transformation is applied and its results are saved into the next matrix. Each individual line of the matrix corresponds to values of one pulse (its PSD). The calculation consists of the division of the signal into M segments which may partly overlap. From each segment [after removal of direct components and by calculating a window (Figure 7)] the middle value of the square of the normalized and amplitude spectrum is calculated. The results for each segment are averaged and deflections made by the used window are removed. This is called the Welch method of modified periodograms [5]. A simplified schema of the calculation is shown in Figure 7.

From the already made signal spectral densities, it was necessary to extract the relevant data and to separate the useful signal from noise or residues formed by surrounding noise or insufficient shielding of an object from surrounding vibrations. This part is quite difficult, because it was not possible to find a suitable routine in Matlab or in the literature concerned with these problems. The question is how to find the position (frequency) of individual points. These points are greatly important for us and directly reflect the real condition of an object. The classic definition of local maximum failed due to the above mentioned problems. It states:

(2) $${\mathrm{maximum}}_{\mathrm{local}}=\mathrm{if}(f_{\left(x-1\right)}<f_{\mathrm{maximu-local}}>f_{\left(x+1\right)})$$

where:

maximum_local_: is a local maximum position
*f*_(x)_: is a value of a function at point *x*

Therefore, it was necessary to find a different method. Human perception of this problem meant great inspiration. When looking at the graph of the power spectrum density [Figure 4(c)], the individual maxima are obvious. After some simplification it is possible to say that perception of individual extremes is due to their position with respect to other values. Even though, the amplitudes of two sharp local maxima which are next to each other are very high, it is possible to ignore them, although if the local maximum is isolated and it has a significantly lower amplitude than the global maximum, it is perceived as sharp. Obtaining relevant points from the PSD is crucial. It is only their position which guarantees correct learning by the neural network or regressive detection and marking of the result. Nowadays, there is a “flag” assigned to each local maximum which states for how long it is valid. In the future, the algorithm will be extended by the option of working in narrow zones and choosing of local maximum more accurately than now. The value of flag is incremented in the case when:

(3) $$↑n_{\mathrm{flag}}=\mathrm{if}(f_{\mathrm{PS}(i)}>f_{\mathrm{PS}(i-1)})$$

Then the position of a local maximum must fulfill the following criterion:

(4) $${\mathrm{maximum}}_{\mathrm{lokal}}=\mathrm{if}(f_{\mathrm{PS}(i)}>f_{\mathrm{PS}(i+1)}\;\mathrm{AND}\;n_{\mathrm{flag}}>n_{\mathrm{set}})$$

where:

n_flag_: actual value of variable n which indicates the operating period of a given maximum
*f*_PS(i)_: is a value of a function (curve PS) in point *i*

Individual results are saved again into a rectangular matrix. With regard to the fact that the filtered spectrum has a number of irrelevant local maxima (the noise component cannot be neglected), it is necessary to apply a final modification which means removal of all local maxima that do not fulfill the criterion:

(5) $$\begin{array}{c} {\mathrm{maximum}}_{\mathrm{lokal}(i)}>\rho \\ \rho ≅0.07\cdot {\mathrm{maximum}}_{\mathrm{global}} \end{array}$$

where:

ρ: constant derived from the global maximum (optional)
*maximum_global_*: value of global maximum from a given PS pulse

**Figure 8.**
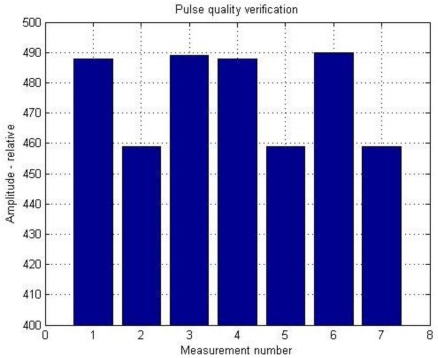
Verifying of pulse’s quality by regressive control of their major frequencies maximums derived from PS.

For verifying whether individual pulses are at least a little similar, the values of global maxima of certain calculations (under the same conditions) are for better transparency arranged next to each other. The final result is a graph (Figure 8).

Furthermore, it is necessary to add that values of frequencies and amplitudes are only relative. By recalculating them it is possible to obtain real frequency and amplitude values. However, this is not necessary for this analysis and it would be more difficult to calculate. Therefore, the values on the axis do not have units (only their relative values are important).

**Figure 9.**
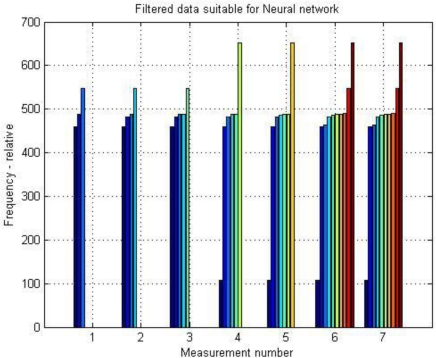
Input data for the neural network. Numbers of major values corresponding to individual points are present in each measurement.

The values from each pulse that are adjusted in this way are saved into a file and presented to the neural network as a teaching pattern or as useful data for detection. The StatSoft program from Statistica was used in order to realize the neural network. This analytical software is primarily intended for data mining, thus for obtaining potentially useful data from a data file. It uses the options and potential of neural networks. Data which are suitable for learning are propounded to the network. The network will choose a given method (back propagation, method of joint neurons, Levenberg-Marquard’s method and so forth). Learning is basically setting of weights of individual neurons. Then the examples of data are given to the network and it determines by certainty the origin of these data or competence to some whole. Graphic representation of input data for the neural network is seen in Figure 9 and some values which are suitable for determining the neural network are in Table 2. All values refer to one series of measures on equal terms.

## 5. Testing and Evaluation of the Proposed Solution

**Figure 10.**
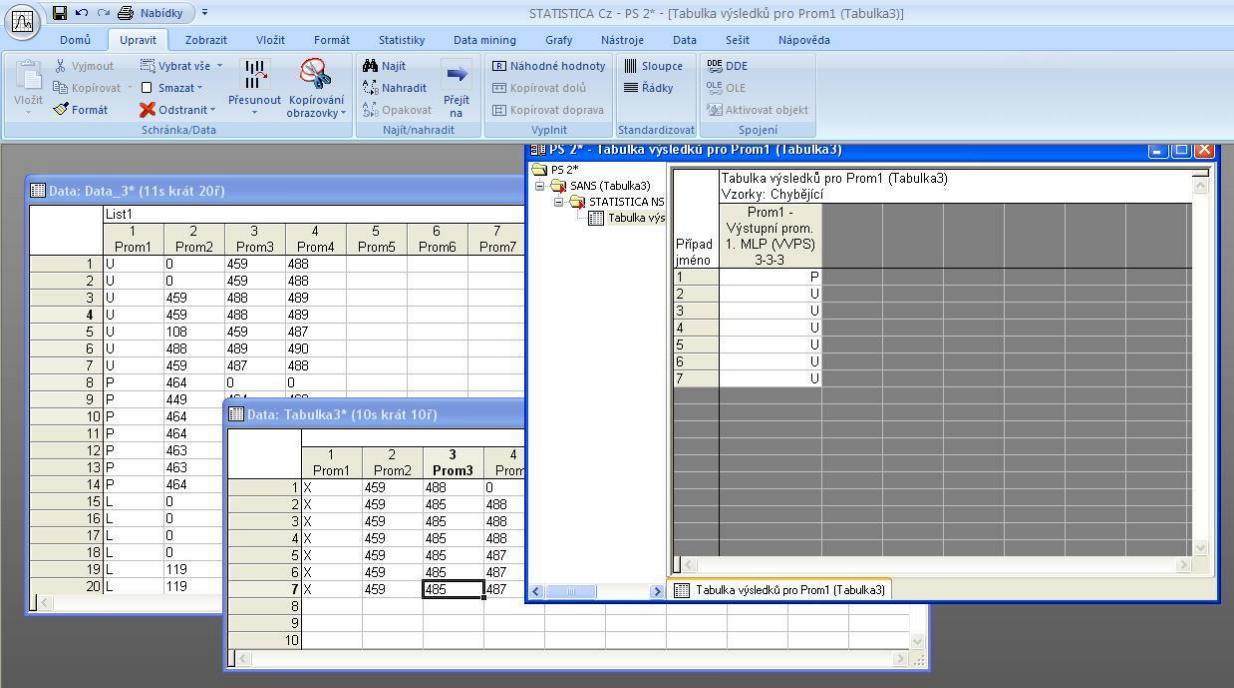
The Statistica program environment and an example of input and output values.

There are a couple of major values in each measurement which correspond to individual points in the signal’s spectrum. In this way the networks which always represent some condition of the investigated object are given. It must be specified in each measurement to which condition it belongs. Samples of measurement (one or more) are presented for detection to the taught network. This detection tries to assign them retroactively to the given condition of an object. For example, the recognition of an individual on the basis of analysis of his/her voice. Each person has a certain voice spectrum, thus the position of local maxima is individual. If the spectral analysis is done from the individual’s voice, the trained neural network would be capable of assigning certain samples to the persons. In our case, the success of regressive assigning of condition samples to which it belongs is in the range of 70–80%. This value depends on the quality of measurement and the inner network state, or how the network is trained. As an illustration, in the right window of Figure 10, there are results of passed analysis of input data and as we can see, the network determined the state of the system with an accuracy of 85.71% (which means that 6 results from 7 were correct). On the image (Figure 10) it is possible to see environment of the Statistica program. The window called DATA_3 contains the training data group. The window called Table 3 also contains the test data group. Finally the last window shows the success of the assignment of testing data to the original group. Training data are marked by the prefixes U, P, L which characterize the condition of an object, thus its inner arrangement (simulation of inner defects). The testing group obtains independent data which were not used for training and serves as an input for the trained neural network. These data were measured on equal terms (prefix U), thus we believe that the network assigns all values to prefix U. It is possible to verify the results in a window and to find out that except one result all of the values are correctly assigned. A first value is incorrectly assigned to other condition of an object (prefix P).

For relevant state detection of an examined object it is necessary to perform several measurements at a row and evaluate them as whole. From our tests it is obvious that a series of 7 measurements is sufficient. Between individual measurements there is always 2 s delay (Figure 3) and so it is necessary to count for 14 s for the whole measurement time. For the reasons given we can assume our solution meets the target of the application field, for example, for processes with slow state changes (on the order of tens of seconds).

## 6. Conclusions

The purpose of this project was to verify whether it is possible to find out the condition of a diagnosed device (presence of inner defects or critical conditions) on the basis of analysis of its vibration power spectrum (acoustic emissions). It is clear from the measurement results that in the frequency area there are some similarities. Another processing of results with the assistance of a neural network can be performed. The condition of an object may be found and whether some critical condition or breakdown has occurred. Correctness of the assignment of individual samples to given conditions is nowadays at least 80%. This number is partly dependent on the data folder which is designed for training the neural network. Therefore, it is necessary to have these operational, critical or breakdown conditions well monitored. By future development of filters and modification of computational algorithms it will be possible to increase the success rate and identify with certainty the condition of an object without requiring a shut-down or by interfering in other ways into its inner structure. Therefore, this method will be useful in places where it is impossible or economically inefficient to shut-down the performance due to preventive maintenance or replacement of device or its parts. For example, this is a continuous steel casting device (losses of hundreds of thousands of $200 tons of steel costs 200 × $2,000 = $400,000 [7]), cracks in pipes and fluid leaks (thousands or hundreds of thousands) or inconsistency in an engine’s operation (price of a new engine), *etc.*

## Figures and Tables

**Table 1.** Summarized comparison of several relevant methods for internal defect detection.

**Emission source**	**Mode**	**Scan type**	**Field of application**					**Solution type**
			**Detection of forthcoming boundary states**	**Detection of boundary states (cracks)**	**Crack position determination (2D)**	**Complexity of application**	**Mobile solution**	
Acoustic emission	On-line	Contact	No	Yes	No	Medium-hard	No	HW+SW
Deterministic pulse	Off-line/On-line	Contact	Yes	Yes	Yes	Medium-hard	No	HW+SW
Acoustic emission	Off-line/On-line	Contact less	No	Yes	No	Easy	Yes	SW

**Table 2.** Example of input data for teaching the neural network (seven measurements of one specific condition of examined object).

**Number of measurement**	**Characteristics frequencies of several measurements (measured at same conditions)**			

1	459	488	550	0
2	459	485	488	550
3	459	485	488	489
4	459	485	488	489
5	459	485	487	488
6	459	485	487	488
7	459	485	487	488
